# An American in India

**Published:** 1892-12

**Authors:** 


### AN AMERICAN IN INDIA.

The above is the title of an article in the *Californian Illustrated Magazine*, for September, from the pen of Dr. Joseph Simms, the celebrated physiologist, who has visited every known section of the globe in the interest of the science to the advancement of which he has devoted many a year.

By permission of the Doctor, we are able to quote some passages from the article in question, which we doubt not, will interest our readers.

India, the middle one of the three irregular peninsulas in the south of Asia, has an area of about a million and a half square miles, so that it is larger than the whole of Europe minus Russia, and more than half the size of the United States of America exclusive of Alaska. The population in 1891 was two hundred and eighty-four million six hundred and fourteen thousand two hundred and ten, quite equal to that of Europe, and about double the estimate made by Gibbon of the nations tributary to imperial Rome. All this densely populated country is now more or less directly subject to British rule.

The Himalaya mountains form the northern boundary of India, and the country south of them consists of vast alluvial plains, extending from sea to sea, and rendered productive by irrigation. The climate is thoroughly tropical, except on the hills, and the extraordinary fertility of the land has passed into a proverb.

India seems to have no plants peculiar to itself, but includes species special to Persia, Siberia, China and Arabia.

Rice is not so extensively cultivated as has generally been supposed, millet being the staple food. Wheat and Indian corn are to be seen in almost every district, barley in somep arts, while oats are cultivated only by Europeans by way of experiment. Oil seeds are an important crop both for home use and for export. All the common vegetables and fruits known to us are found here; also spices, among which turmeric and chillies hold the first place. Cotton is the product most grown for export, and jute ranks next to it as a fibre crop. Indigo has been the plant most cultivated by European capital, but its importance is declining. The opium poppy is raised in some places as a government monopoly; in other places it is subject to restrictions, and throughout the greater part of the country it is entirely prohibited. The principal fields of this culture occupy five hundred and sixty-two thousand acres, chiefly in Bengal. Most of the opium goes to China, the revenue therefrom amounting to several million dollars. The cultivation of coffee has long been practiced by the natives, but that of tea, which is rapidly increasing, is recent, and due wholly to European enterprise. There are many valuable medicinal plants, as those from which we have the cinchona bark and croton oil; but it would be almost an endless task even to enumerate the varied productions of this fertile land.

The forests were in some danger of being exterminated through the recklessness of timber cutters, charcoal burners and others. But they are now protected by the government, and the chief exports from this source are teak, lac, bamboo canes, caoutchouc and other gums. The date and cocoanut palms yield much of the native food, and from the latter, arrack, an intoxicating drink, is obtained. Tigers, leopards, jackals, wolves, hyenas and wild boars abound in the country, the first two being very destructive to men and cattle. The elephant, naturally wild, is tamed and made useful.

The domestic bovines are used for farm labor and draught. The wild species known as buffaloes or bisons is dangerous, and keeps away from human dwellings. Most of the gentler and smaller quadrupeds familiar to ourselves are natives here. Quadrumanous animals are numerous, as are also the serpent tribes, the bites of some of which are rapidly fatal. Insect tribes are innumerable. Annually, about twenty-five thousand persons are killed by wild beats in British India, about one thousand one hundred and fifty of whom perish from the stings and bites of scorpions, lizards and mad dogs.

The natives of India are very superstitious and believe in a host of unreasonable traditions of the past and signs of the future. At Delhi, we were shown a stone slab, bearing the impress of two feet, said to be those of the great founder and teacher of their religion. At Ghat, in Benares, is a slab of marble, with an imprint said to have been made by the feet of Vishnu, and at certain seasons of the year people flock to this place to worship these foot prints. At Bhaurava Ghat, in Benares, there is an image in stone, the face being covered with silver. It represents Siva in terrific form; the people who visit it present offerings of sugar dogs; and a Brahman waves a fan of peacock feathers over the devotees to protect them from evil spirits.

Great sanctity is attached to the Kad-jakada ape with red face and black beard; it is regarded as an incarnation of Siva. One variety of the ox and cow called the humped zebu is considered sacred and treated with reverence. Every temple has a sacred bull, and the cow is honored with the title of "mother of the gods." The tradition is that Brahma created the Brahmins and the cow at the same time.

Another sacred creature is the Coromandel eagle, which is considered as an incarnation of Doorga. Ravens and rooks are believed to be receptacles of human souls that have left the body.

Everywhere among uneducated natives we meet with gross and childish superstitions, but perhaps the strangest and most peculiar of all the superstitious usages we witnessed was that of the mouth lock. It is an instrument somewhat like a large safety-pin, generally of silver, but sometimes of gold, brass or copper. The pin is run through both cheeks, behind the corners of the mouth, and between the teeth. The cheeks are drawn so closely together that the mouth is kept constantly open. When a Hindoo desires some special benefit from the gods, it is usual to make a vow, and he puts on the mouth lock in token of his vow, which implies entire abstinence from food and complete silence. When the end has been answered, the devotee goes to the shrine to take it off and place it in the receptacle appropriated for receiving the offerings of pilgrims. Fifty such locks may be given up at one temple in one year. Tirupati is a place where thousands are surrendered.

They are afterwards sold by auction as old silver, and the money realized goes to the benefit of the temple. They are worth about ten to fifty cents apiece.

The Parsees, who are most numerous in Bombay, hold the highest place among the native communities, not on account of superior numbers, but because of their wealth, intelligence, genius for trade, and munificent charities. They are descendants of those fire-worshippers who were driven from Persia about a thousand years ago by the conquering hosts of Mohammedans from Arabia ; and as they seldom intermarry with the other races of India, they continue physically distinct, bearing much greater resemblance to Europeans.

One of the most singular practices of the Parsees is their mode of disposing of the dead. If repulsive to our minds, it must be admitted to have sanitary advantages. Outside a town containing Parsee residents, there are circular buildings called "Towers of Silence," in which the corpses are placed and devoured by hundreds of vultures, always on the watch. At a little distance from Kurrachee are two such towers. They are stone buildings, without windows or roof, about twenty or thirty feet high, and from forty to fifty feet in diameter, with a door for entrance. Before placing a corpse inside the tower, it is laid on a stone outside for the inspection of a dog which is supposed to be able to indicate the state of the departed soul. If the dog looks at the face of the dead, the soul is supposed to be in heaven ; if he does not the spirit is said to be among the lost. Four dogs are kept near the tower of silence to give these intimations. When by the dog test the spiritual condition of the departed has been ascertained, the nude body is placed on the stone inside the tower, and in less than half an hour the vultures have stripped off every particle of the flesh. The bones are then mixed with quicklime, so that in a few days not a vestige remains of what was a human body. No tombstone is erected to commemorate the departed. We understood that the reason of this singular mode of disposal of the dead is the desire not to pollute any of the elements of nature with the impurities of decay. At Bombay there are five such towers of silence.

On the bank of the Jumna lies the ancient city of Agra, believed by the natives to have been the place of the sixth incarnation of the god Vishnu, and here can be seen the Taj Mahal—the crown of empires—one of the largest of all known mausoleums, unequaled throughout Asia for the beauty of its design and the perfection of its finished execution. Here repose the remains of Arjmased Banu, signifying the pride of the palace, the favorite wife of the Emperor Jahan. She died in 1629, at Burkanpur, in the Deccan. Her mausoleum is said to have occupied twenty thousand men for seventeen years in building and to have cost fifteen million seven hundred and forty-eight thousand dollars. Austin de Bordeaux, a Frenchman, is supposed to have been the architect. Its diameter is one hundred and eighty-six feet and it stands on a raised platform of white marble eighteen feet high and three hundred and thirteen feet square, with a tower of white and black marble at each corner.

The Taj Mahal is the most majestic and enchanting monument on earth. It is a grand representation of the sacredness of human affection. In memory of his beloved wife a powerful monarch could not build more costly and beautiful than this anaglyph in snowy marble, glittering as myriads of diamonds beneath a rich flood of Oriental sunlight. Its rectangle of arabesques, its aesthetically carved cupola, and its graceful minarets, prodigies of artistic design and finish, have never been equalled in grandeur by any structure erected in honor of the dead.

